# Effect of Dentin Biomodification on the Survival of Resin Composite Restorations: An Umbrella Review

**DOI:** 10.1016/j.identj.2026.109446

**Published:** 2026-02-18

**Authors:** El Alaoui Nihal, Chala Sanaa, Ghoul Sonia

**Affiliations:** a International Faculty of Dental Medicine, Research Center of Health Sciences (CReSS), International University of Rabat (UIR), Sala-Al Jadida, Morocco; b Faculty of Dental Medicine, Mohammed V University in Rabat, Mohamed V Military teaching hospital, Rabat, Morocco; c Laboratory of Biostatistics, Faculty of Medicine and Pharmacy, Clinical and Epidemiological Research, Mohammed V University in Rabat, Rabat, Morocco

**Keywords:** Dentin, Composite resins, Biomodification, Durability, Collagen, Matrix metalloproteinases, Systematic review

## Abstract

**Introduction and aims:**

Achieving durable adhesion of composite-resins to dentin remains a key challenge in restorative dentistry, highlighting the need for biomodification strategies. Therefore, this umbrella review aims to systematically overview dentin biomodifiers and evaluate their influence on the durability of composite-resin restorations.

**Methods:**

Literature search was conducted in PubMed, Scopus, and Web of Science up to October 2024, focusing on three concepts: dentin, biomodification, and bond strength (PROSPERO: CRD42024588804). The corrected covered area (CCA) was calculated to evaluate overlap among the included systematic reviews (SRs).

**Results:**

Among 486 identified papers, 9 SRs were selected including 7 meta-analyses. Twenty biomodification agents were identified and classified into natural, physical and chemical agents. These agents were assessed in 51 different setups with micro-tensile bond strength used in 68.62% of them. Timing of bond strength assessments varied from immediate to a 36-months period. As natural agents, Grape Seed Extract and chitosan improved bond strength after long-term aging, while induced riboflavin improved the short and medium-term periods only. Among all categories, the most studied biomodifier was Non-Thermal Atmospheric Plasma, a physical agent, which consistently enhanced resin-dentin bonding over time. However, chemical approaches did not show promising bond strength results at some exceptions. Sodium hypochlorite (NaOCl) and hypochlorous acid both immediately decreased bond strength. Overlap among reviews was slight (CCA = 1.52 %).

**Conclusion:**

Various biomodifiers show promise for enhancing adhesion and durability of composite-resin restorations, with their combinations potentially offering synergistic effects.

**Clinical relevance:**

Systematically identifying and characterizing dentin biomodifiers within their limitations lays the groundwork for evidence-based frameworks that guide dentists and shape future research. At present, physical scrubbing and air abrasion are the only readily available dentin biomodification techniques. Riboflavin and chitosan show promise as effective natural and affordable agents; however, their clinical use needs regulatory approval. In contrast, NaOCl should be avoided as it compromises composite restoration’s durability.

## Introduction

Durability of composite restorations refers to the resin composite’s ability to maintain its mechanical integrity, aesthetic qualities and a stable adhesive interface, characterized by long-term bond strength, despite the challenging conditions of the oral environment.^1^^,^^2^ This durability is critically influenced by both sides of the resin-dentin interface, namely the dentin substrate and the resin-composite material.

Composite resins represent the most commonly used materials in dental restorative procedures. Hence, they must withstand occlusal forces, fatigue, chemical degradation, and moisture exposure. Despite significant material advances, key limitations persist for their durability.^3^^,^^4^ In fact, polymerization shrinkage, water sorption, and incomplete monomer conversion lead to induced stress and degradation, reducing mechanical stability.^5^^,^^6^ These shortcomings, combined with the passive nature of most conventional materials, highlight the growing need for restorative systems with bioactive properties, capable of interacting with the surrounding environment to support long-term restoration wear.^7^

The dentin substrate, on the other hand, possesses several characteristics with significant implications for the bonding’s success as it consists of approximately 70% hydroxyapatite crystals and 30% hydrated organic matrix. This matrix features a network of unmineralized collagen fibres oriented perpendicularly, through which dentinal tubules pass, and enzymes mainly matrix metalloproteinases (MMPs). However, acidic conditions, such as those induced by caries or etching, initiate collagen breakdown through MMP activation while also promoting demineralization through the dissolution of mineral ions.^8^^,^^9^ These features combine to compromise the structural integrity of dentin and challenge the formation of a stable three-dimensional polymer–collagen network, known as the hybrid layer.^10^

Therefore, dentin biomodification offers a promising solution to these problems all together. It can be defined as a biomimetic strategy mediated by bioactive agents, that reinforces collagen and regulates biodegradation rates by locally altering the biochemistry and biomechanical properties of dentin. Hence, dentin biomodifiers, either applied to dentin surface or incorporated into restorative material, target the resin-dentin interface which is the critical weak point in restoration integrity. They act on stabilizing collagen via collagen cross-linking, enzyme inhibition and dentin regeneration through reinforcement of the mineral phase.^11^^,^^12^ Thereby, the resin-dentin bond is preserved over time increasing the durability of composite-resin restorations. Early evidence suggests its efficacy, indicating that including biomodifiers into restoration protocols may represent a paradigm shift in adhesive dentistry.^13^ However, the lack of a clear classification of dentin biomodifiers may result in their misuse or unintentional use in clinical practice, underscoring the need for evidence-based frameworks to define and contextualize their application. Therefore, this umbrella review aims to provide an overview of the various existing dentin biomodifiers and to evaluate their influence on the durability of composite-resin restorations over time.

## Material and methods

This umbrella review was conducted in accordance with the Preferred Reporting Items for Systematic Reviews and Meta-Analyses (PRISMA) statement checklist 2020. The protocol was registered in the international prospective register of systematic reviews (PROSPERO: CRD42024588804).

### Research question

The proposed research question for this study was: 'How do biomodifiers applied to dentin influence the bond durability of resin composite restorations?'. This research question was constructed based on the following PICOS elements for Population (P): resin-composites restorations; Intervention (I): application of biomodifiers on dentin surface prior to restoration; Comparators (C): resin-composite restorations with no prior dentin pretreatment; Outcomes (O): bond durability of resin-composites; Study design (S): systematic reviews of in vitro studies, with or without meta-analysis.

### Search strategy

An electronic search was conducted by two authors (N.E.A, S.G) in three databases: PubMed, Scopus, and Web of Science Core Collection, on October 2024. The search strategy cantered around 3 concepts: dentin, biomodification and bond strength; and was appropriately adapted for each database (Appendix A). No restrictions on date or publication language were applied.

### Eligibility criteria

Systematic reviews (SRs) of in vitro studies, with or without meta-analysis evaluating bond durability of direct resin composite restorations to dentin of adult human teeth after pretreatment with biomodifiers, were included. Systematic reviews focusing on teeth with structural anomalies or on materials other than composite resins and their adhesives (eg, sealants, luting cements or fibre posts) were excluded (Table A.4) (Appendix A).

### Selection process

Search results were imported into Zotero, and duplicates were manually removed and double-checked. Titles and abstracts were screened. Studies with unavailable abstracts or abstracts with insufficient information were retained for full-text review. Full-text screening was independently performed in duplicate by two authors (N.E.A, S.G). In cases of missing information, the corresponding authors were contacted by email, with a follow-up message sent after one week. Studies were excluded if the missing data could not be obtained. Any discrepancies at any stage of the selection process were discussed with a third author (S.C) until consensus was reached.

### Data collection

A standardized data extraction sheet was developed by all authors and refined based on pilot testing. Two independent reviewers (N.E.A, S.G.) extracted data in duplicate, as reported in the SRs. The data extracted included PICO items, objectives, quality assessment tools, adhesive strategies, ageing strategies, and number of included studies in the systematic review and in the meta-analysis if applicable. PICO elements were extracted, with consistency ensured through strict adherence to the research question and pre-defined eligibility criteria. Regarding quality assessment, the specific tools utilized within the included SRs were recorded for descriptive purposes. Adhesive strategies were initially extracted as reported and subsequently harmonized, when necessary, according to their bonding mechanism. Specifically, 1-step and 2-step self-etch systems were consolidated under the 'self-etch' category, while 2-step and 3-step etch-and-rinse adhesives were consolidated under 'etch-and-rinse' category. Universal adhesives were allocated to one of these 2 categories based on their mode of application.^14^ Ageing strategies were extracted as reported and found to be consistent across SRs.

Biomodification agents were extracted and categorized as natural, physical and chemical based on their nature. Bond strength testing time points were extracted and grouped into three periods: short-term (<3 months), medium-term (3-12 months), and long-term (≥12 months). This classification was based on the prevailing reporting patterns in the included SRs; and was adopted to facilitate a standardized comparison and prevent significant data loss.

### Calculation of the degree of overlap

To assess overlap of primary studies across the included SRs, citation matrix was generated using the Graphical Representation of Overlap for OVErviews (GROOVE) and the 'Corrected Covered Area' (CCA) was calculated using the following formula *CCA* = $\frac{N-r}{rc-r\;}$ with *(N)* being the number of included primary studies including duplicates, *(r)* the number of index publications, and *(c)* the number of included systematic reviews.^15^ The *CCA* [%] was interpreted as slight (0%-5%), moderate (6%-10%), high (11%-15%), and very high (>15%) overlap as described by Pieper et al.^16^

### Risk of bias assessment

Two reviewers (N.E.A, S.G) assessed the risk of bias within systematic reviews independently and in duplicate using Risk of Bias in Systematic reviews (ROBIS) tool consisting of three phases: (1) Optional assessment of the systematic review’s relevance, (2) Identification of concerns with the review process including the four domains: study eligibility criteria, identification and selection of studies, data collection and study appraisal, as well as synthesis and findings and (3) Judgement on the risk of bias in the systematic review.^17^ Any disagreements were resolved through consultation with a third author (S.C).

## Results

### Literature search

**Fig. 1:**
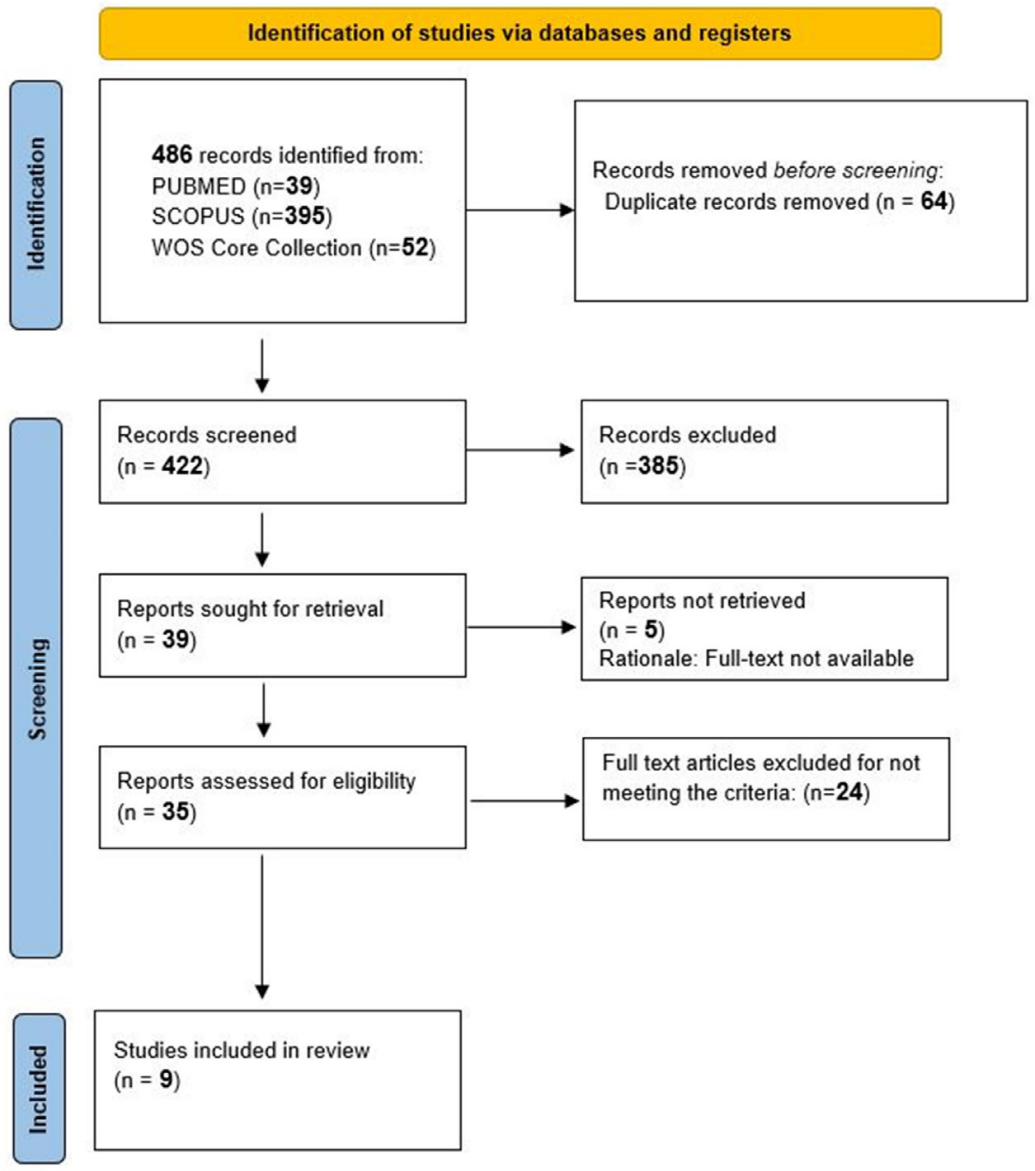
PRISMA flowchart.

A total of 486 articles were initially identified. After removing 64 duplicates, 383 records were excluded after the titles and abstracts were screened. Five reports could not be retrieved as they were not found in full-text. Full-text eligibility was assessed for 34 papers by two independent reviewers (N.E.A. and S.G.). Twenty-five studies were excluded for failing to meet the inclusion criteria, while nine studies were deemed eligible and included in the current overview (Figure 1). Among these selected studies, 175 primary studies were considered including 19 duplicates (Table B.1) (Appendix B).

### Quality assessment

**Fig. 2:**
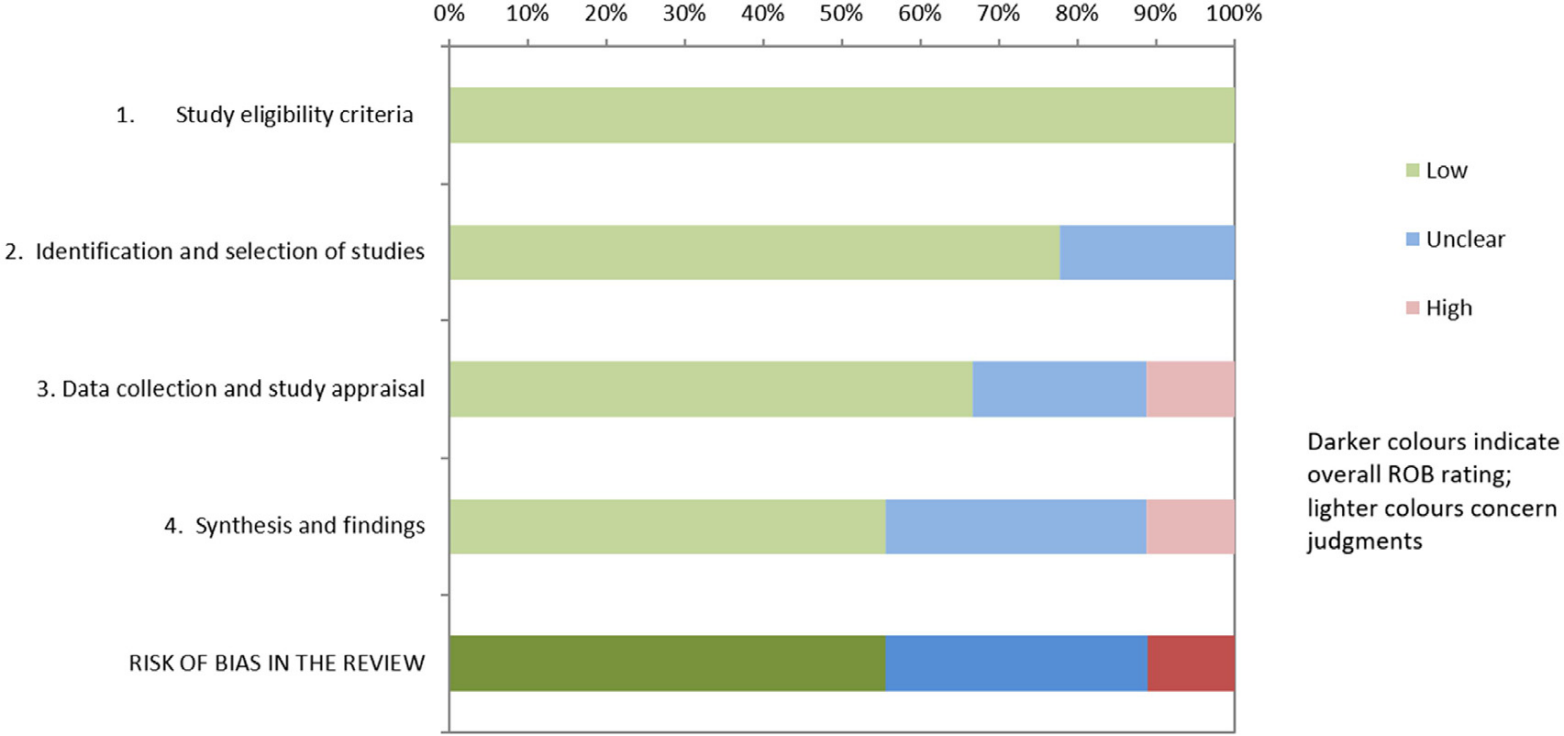
Overall risk of bias of each domain of ROBIS. ROBIS risk of bias assessment across four domains and overall rating. Colours represent judgments: green (low risk), blue (unclear risk), and red (high risk). Darker colours indicate overall risk of bias rating.

The included SRs employed multiple risk of bias assessment tools. Specifically, four reviews employed a customized tool based on previous research, one used the RoBDEMAT tool, one used a quality rating form developed by the Cochrane Collaboration, one applied a custom Cochrane Collaboration tool, one used a modified CONSORT checklist, and one did not use any tool for risk of bias assessment. For this umbrella review, all included SRs were assessed using ROBIS tool (Table 1). Three SR presented low risk of bias for all domains,^18^, ^19^, ^20^ two SR presented unclear risk for one domain only,^21^^,^^22^ three SR showed at least two domains with unclear concern,^23^, ^24^, ^25^ and one SR presented high risk for three domains.^26^ According to the five domains and third phase of the ROBIS tool: 100% of reviews had low risk of bias in study eligibility criteria; 75% of reviews had low risk and 25% had unclear risk in identification and selection of studies; 70% of reviews had low risk, 20% had unclear risk and 10% had high risk in data collection and study appraisal; 60% of reviews had low risk, 25% had unclear risk and 15% had high risk in synthesis and findings and finally; 50% of reviews had low risk, 30% had unclear risk and 20% had high risk in the overall risk of bias in the review (Figure 2). The overlap among the included SRs was assessed, and the CCA was 0.0152 (1.52%).

**Table 1:** ROBIS results.

### Study characteristics

**Fig. 3:**
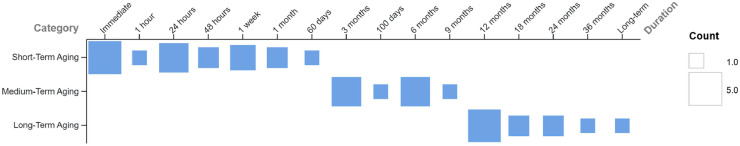
Aging durations among the included SRs. This matrix plot maps aging duration (x-axis) to aging category (y-axis): short-term (<3 months), medium-term (3-12 months), and long-term (≥12 months). Square size represents the number of systematic reviews per duration.

The characteristics of the included SRs were summarized in (Table 2). Among the nine SRs, seven were meta-analyses, including one network meta-analysis.^18^, ^19^, ^20^, ^21^, ^22^, ^23^, ^24^, ^25^, ^26^ Publication years ranged from 2018 to 2024. Seven SRs assessed micro-tensile bond strength (µTBS) either exclusively or among other bonding tests, while Wang et al^19^ focused only on shear bond strength (SBS) and Stasic et al^18^ did not specify the test method used. The timing of bond strength assessments varied, ranging from immediate testing to a 36-month aging period. The testing periods most frequently reported across the studies were: immediate and 12 months, each addressed by five different SRs; followed by 24 hours, 3 months, and 6 months, each reported in 4 different SRs (Figure 3). Five different methods of aging were reported in the SRs. One SR^26^ used water storage alone. Another one^22^ applied distilled water and artificial saliva, while Anumula et al^24^ combined them with thermocycling and Awad et al^21^ combined water storage, thermocycling, and chloramine solution storage. Two SRs^20^^,^^25^ both used artificial saliva, thermocycling, and collagenase ageing, with Hardan et al^25^ also including water storage. Three SRs did not specify the aging method used.^18^^,^^19^^,^^23^

**Table 2:** Characteristics of included systematic reviews.

Authors/Year	Meta-analysis	Primary studies	Biomodification agent	Aging duration	Outcome measured	Conclusion
Alshaikh et al^26^	Yes	9	NaOCl HOCl	24h	Micro-tensile bond strength	Long exposure to deproteinizing agents significantly impairs the bonding of self-etch agents to dentin.
Alves et al^23^	Yes	11	Chitosan	Immediate 6 months 12 months 36 months	Micro-tensile bond strength Shear bond strength	Chitosan appears promising on the bond strength of the adhesive interface when used as a pretreatment on dentin.
Anumula et al^24^	Yes	14	GSE CSE GTE EGCG Baicalein	Immediate 24h 3 months 6 months 9 months 12 months 18 months	Micro-tensile bond strength	The available evidence indicates that GSE is the most efficient natural cross-linker to date, in preserving the BS even after ageing. The other natural cross-linkers did not show such an improvement in the BS after ageing, but it is evident that they may not impair the BS.
Awad et al^21^	No	13	Non-thermal atmospheric plasma	24h 48h 1 week 60 days 12 months	Micro-tensile bond strength	NTAP application could enhance resin–dentin micro-tensile bond strength of etch-and-rinse adhesives or universal adhesives applied in the etch-and-rinse mode
Eusufzai et al^22^	Yes	17	Riboflavin	Immediate 24 h 48h 1 week 1 month 6 months 12 months 18 months	Micro-tensile bond strength	After 24 h of aging and 6 months of aging show improvement in micro-tensile bond strength with the use of riboflavin collagen crosslinker compared to without use of Riboflavin.
Hardan et al^25^	Yes	42	NTAP Laser Ethanol Dentin desensitizers Air abrasion Scrubbing Warm air blow Glycolic acid Ozone Electric current	1 hour 24 hours 1 week 100 days 3 months 6 months 1 year	Shear bond strength Micro-shear bond strength Tensile bond strength Micro-tensile bond strength	Application of non-thermal atmospheric plasma, ethanol-wet bonding strategy are recommended
Stasic et al^18^	Yes	17	NTAP	Immediate Long term	Nsp	NTAP has significant short- and long-term effects on adhesive-dentin bond strength when compared to control treatments.
Wang et al^19^	Yes	40	Erbium laser	Nsp	Shear bond strength	Both Er:YAG and Er,Cr:YSGG lasers improved dentin bond strength compared to blank controls.
Zhang et al^20^	No	12	DMSO	1 month 3 months 6 months 12 months 24 months	Micro-tensile strength	Evidence proves the promotion of DMSO wet bonding on the long-term dentin bonding stability of etch-and-rinse system. However, its effect on self-etch system remains controversial and requires more evidence to prove. DMSO can act as an outstanding solvent for incorporating natural plant extracts to obtain a synergistic effect to optimize dentin bonding.

Characteristics of included systematic review and their authors conclusions.

CSE, cocoa seed extract; DMSO, dimethyl sulfoxide; EGCG, epigallocatechin gallate; GSE, grape seed extract; GTE, green tea extract; HOCl, hypochlorous acid; NaOCl, sodium hypochlorite; NTAP, non-thermal atmospheric plasma; Nsp, not specified.

### Outcomes

***1. Bond strength tests and adhesive strategies***

**Fig. 4:**
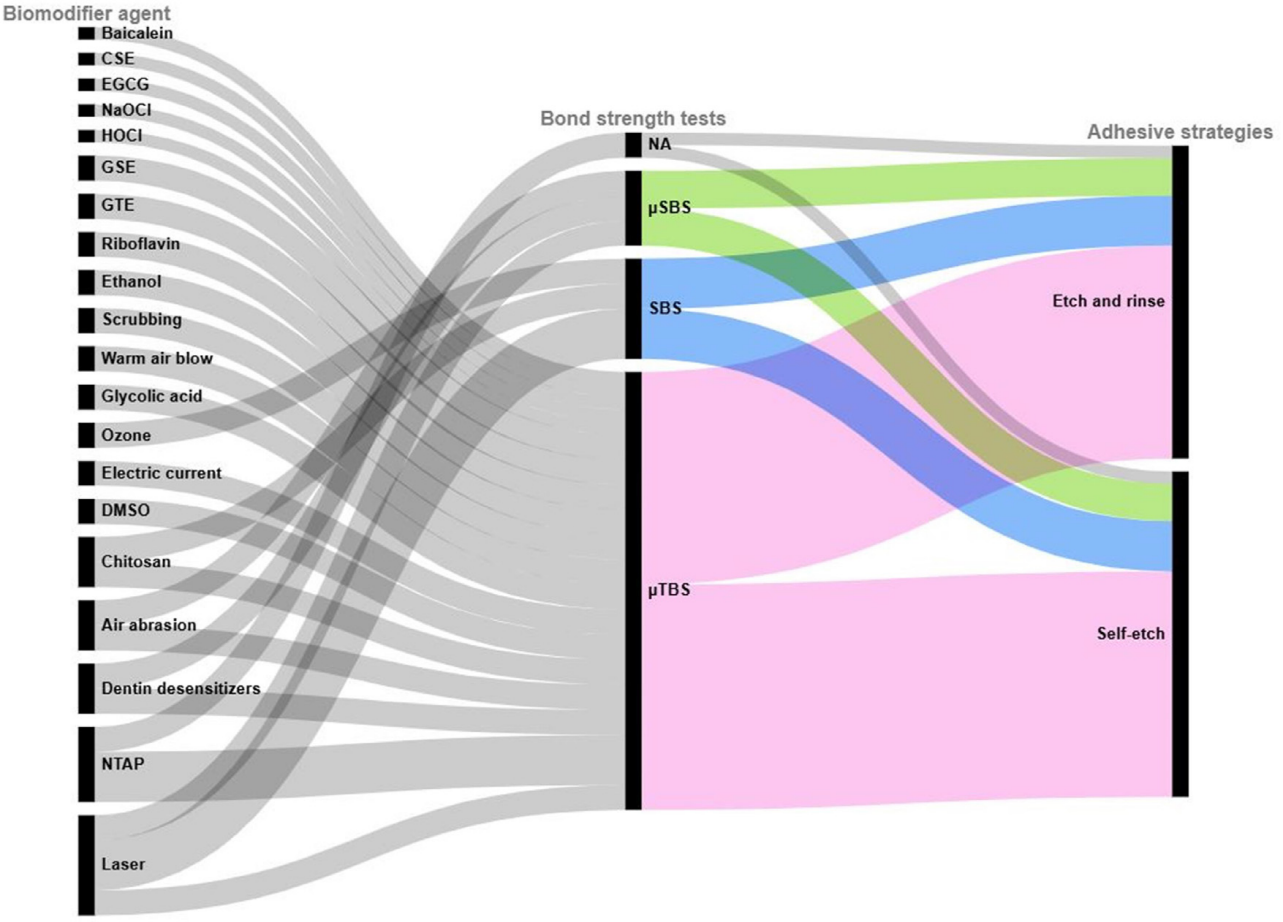
Biomodifier agents, bond strength tests and adhesive strategies. This alluvial diagram illustrates the relationships between biomodifier agents, bond strength tests, and adhesive strategies. Each rectangle represents a category within these dimensions, with its height proportional to its occurrence. The curved lines (flows) indicate connections between categories, with their width reflecting the strength of the correlation. CSE, cocoa seed extract; DMSO, Dimethyl Sulfoxide; EGCG, epigallocatechin gallate; GSE, grape seed extract; GTE, green tea extract; NTAP, non-thermal atmospheric plasma; SBS, shear bond strength; TBS, tensile bond strength; μSBS, micro-shear bond strength; μTBS, micro-tensile bond strength.

The relationships between the three dimensions of biomodifier agents, bond strength tests, and adhesive strategies, based on data extracted from the included SRs, are mapped in Figure 4. Each rectangle represents a unique category within one of these dimensions. The height of each rectangle is proportional to the number of connections it shares with the other categories. The curved lines connecting the dimensions indicate shared occurrences, with their width reflecting the strength of the correlation. Fifty-one experimental setups were identified across the primary studies within the included SRs. LASER was evaluated across the broadest spectrum of experimental varieties, as it was assessed in 8 different setups followed by non-thermal atmospheric plasma (NTAP) assessed in 6 setups. As for the testing methods, µTBS (pink) was reported in 35 experimental setups, accounting for 68.62%, followed by SBS (blue) in 8 setups (15.68%). Micro-shear bond strength (µSBS) (green) was used in 6 setups, representing 11,76% while 2 did not specify the test used, accounting for 3,92% (grey). Moreover, the biomodifier agents were investigated under both adhesive strategies, with a nearly equal distribution between etch-and-rinse and self-etch systems, adopted by 25 and 26 setups, accounting for 49,01% and 50,98% respectively.

***2. Biomodifiers effects on bond strength***

Twenty biomodification agents were identified and categorized based on their type into 3 main categories: natural agents, physical agents and chemical agents. Table 3 summarizes the effects of biomodifiers from the included meta-analyses only by evaluation period. Significant outcomes (*P* < .05) are marked as positive (green), negative (red), or controversial (yellow), while non-significant findings are shown as no effect.

**Table 3:** Biomodifiers effects from included meta-analyses.

***2.1. Natural agents***

***2.1.1. Baicalein***
  Baicalein was evaluated over short and medium-term durations in one SR.^24^ The results favoured Baicalein use compared to untreated control, despite variations in its concentration.
***2.1.2. Chitosan***
  Chitosan and chitosan nanoparticles (CS-NPs) were evaluated on the immediate, medium-term and long-term periods in one SR with meta-analysis.^23^ Prior use of chitosan and CS-NPs improved immediate bonding to dentin when applied under the self-etch mode, with Standard Mean Difference (SMD) of −0.75, favouring chitosan and supported by low heterogeneity (I² = 35%). For medium- and long-term ageing, bond strength improved under the etch-and-rinse mode (SMD = -1.24), increasing durability of dentin bonds with moderate heterogeneity (I² = 50%). No improvement was seen with the self-etch mode over time.
***2.1.3. Cocoa seed extract (CSE)***
  The effect of CSE on bond strength over both medium and long-term periods was evaluated in one SR with meta-analysis.^24^ The results showed no significant improvement in bond strength after CSE pretreatment compared to no treatment (*p* = 0.23).
***2.1.4. Grape seed extract (GSE)***
  The effect of GSE on medium and long-term bond strength was evaluated by one SR and meta-analysis.^24^ On the medium-term, GSE showed a beneficial impact on bond strength with Mean Deviation (MD) of 8.29 favouring GSE to untreated control, with high heterogeneity (I² = 96%). At the long-term period, GSE significantly improved the durability of the bonding (MD = 13.88), with moderate heterogeneity (I² = 46%).
***2.1.5. Green tea extract (GTE)***
  The effect of GTE on bond strength over medium and long-term periods was evaluated by one SR with meta-analysis.^24^ While GTE may not have shown significant results for the medium-term ageing due to high heterogeneity and *p*-value, it has exhibited an appreciable improvement in bond strength in the long-term period compared to no treatment.
***2.1.6. Epigallocatechin Gallate (EGCG)***
  The effect of EGCG on bond strength over time was evaluated by one SR with meta-analysis.^24^ No significant improvement in bond strength was observed compared to no treatment (*p* = 0.95).
***2.1.7. Riboflavin***
  Riboflavin was evaluated in one SR with meta-analysis.^22^ After short-term and medium-term of aging, an improvement in micro-tensile bond strength was observed with the use of riboflavin, with MD values of −8.49 and −10.23 and I^2^ of 89% and 96% respectively. Additionally, photoactivated riboflavin showed better results in enhancing micro-tensile bond strength compared to non-photoactivated riboflavin.

***2.2. Physical agents***

***2.2.1. Non-thermal atmospheric plasma (NTAP)***
  NTAP was evaluated by three SRs^18^^,^^21^^,^^25^ and their findings were consistent. The first SR^21^ concluded that NTAP could enhance resin-dentin μTBS when used under the etch-and-rinse mode without specifying duration. The second review,^25^ including a meta-analysis, showed that while NTAP may not have improved bond strength immediately after application, it did enhance long-term results as SMD is of −0.82 with I^2^ = 75%. The third systematic review,^18^ which included a meta-analysis, evaluated both Argon and Helium-based plasmas. The overall results showed that NTAP had significant short- and long-term effects on adhesive-dentin bond strength compared to control treatments, with SMD values of 1.92 and 3.28 respectively and I^2^ of 97% for both periods. Specifically, He-NTAP demonstrated improved bond strength in both the short- and long-term, while Ar-NTAP showed significant short-term effects only.
***2.2.2. Scrubbing***
  The scrubbing technique was evaluated by one SR with meta-analysis.^25^ µTBS was assessed, showing significant improvement in bond strength for both immediate and medium-term aging after scrubbing was applied, compared to untreated dentin.
***2.2.3. Air abrasion***
  Air abrasion was evaluated in one SR and meta-analysis.^25^ µTBS was assessed after pretreatment on the short-term period. The SMD was −0.75 favouring air abrasion to no treatment, with a 95% confidence interval (CI) ranging from −1.79 to 0.29 and high heterogeneity (I² = 92%).
***2.2.4. LASER***
  LASER pretreatment for dentin was evaluated by two SRs,^19^^,^^25^ one with a meta-analysis and the other with a network meta-analysis, both reporting varying findings. The first SR^25^ assessed bond strength across all time periods. It showed an overall trend of decreased bond strength compared to no treatment, though this was not statistically significant (*p* = 0.06). However, bond strength at the immediate term was significantly reduced after LASER application, with a moderate heterogeneity of 56%. The second SR^19^ focused on dentin SBS after pretreatment with Erbium LASERs. Both Er:YAG and Er,Cr:YSGG LASERs improved bond strength, with Er:YAG being more effective. Er:YAG LASER combined with acid etch-and-rinse treatment showed the best results, outperforming etch-and-rinse alone.
***2.2.5. Warm air blow***
  Air-blowing with warm air was evaluated by one SR,^25^ that assessed its use in the immediate and medium-term periods.
***2.2.6. Electric current***
  Electric current was evaluated by one SR with no reported outcomes on bond strength.^25^
***2.2.7. Ozone***
  Ozone was evaluated by one SR.^25^ It was used as dentin disinfectant for its effect on short-term bond strength, with no outcomes reported.

***2.3. Chemical agents***

***2.3.1. Dentin desensitizers***
  Dentin desensitizers were evaluated by one systematic review and meta-analysis.^25^ They were found to deteriorate bond strength both immediately after application and over time (SMD = 0.96). This result was statistically significant but present high heterogeneity (I² = 80%).
***2.3.2. Dimethyl sulfoxide (DMSO)***
  DMSO was evaluated by one SR,^20^ showing that DMSO wet bonding positively impacts the long-term dentin bonding stability of the etch-and-rinse system, although not in a dose-dependent manner. Its effect on the self-etch system remains controversial. Additionally, DMSO served as an effective solvent for incorporating natural plant extracts, potentially enhancing dentin bonding through synergistic effects.
***2.3.3. Ethanol***
  The ethanol wet bonding technique was evaluated by one SR with meta-analysis,^25^ showing an improved immediate bond strength after application compared to no treatment control (SMD= -1.41) with low heterogeneity.
***2.3.4. Hypochlorous acid (HOCl)***
  The effect of HOCl, a deproteinizing agent, was assessed by a SR and meta-analysis,^26^ on dentin bonding in self-etch mode. High concentrations of HOCl significantly reduced μTBS values, and prolonged exposure to the agent further compromised bonding to dentin. In addition, 2 steps self-etch adhesives demonstrated higher bond strength than 1 step self-etch adhesives when applied to deproteinized dentin.
***2.3.5. Sodium hypochlorite (NaOCl)***
  The effect of NaOCl was evaluated by a SR and meta-analysis^26^ in self-etch mode. Pretreatment with NaOCl led to low μTBS values compared with non-treated surfaces. Also, NaOCl solutions exhibited more significant adverse effects on bonding to dentin compared to HOCl, despite both being deproteinizing agents.
***2.3.6. Glycolic acid***
  Glycolic acid was evaluated by one SR^25^, assessing its effect on bond strength. However, an absence of evidence was noted, as no outcomes were reported.

## Discussion

This umbrella review evaluated the effect of various biomodifiers on the adhesion of composite-resins to sound human dentin, based on the results of published systematic reviews of in vitro studies, most of them being published after 2021. It has also presented a slight overall overlap (CCA = 1.52%) meaning that only a small number of studies were repeated across the systematic reviews.^16^ This suggests minimal redundancy and a broad evidence base, which increases robustness of the overall conclusions. Nonetheless, pairwise comparisons revealed very high overlap (CCA = 57, 9%) between two particular SRs,^18^^,^^21^ both evaluating NTAP (Figure B.1) (Appendix B). This could bias the true pretreatment effect and should be considered when interpreting NTAP results. Meanwhile, the quality assessment of the included reviews revealed a low to medium risk of bias in the first ROBIS domain only, with increasing concerns in later review stages, suggesting that higher bias may have been introduced, particularly during the synthesis and overall appraisal. Consequently, the conclusions remain generally reliable but should be interpreted with measured caution. In addition, categorization of bonding intervals into short-, medium-, and long-term periods prevents significant data loss. However, it is acknowledged that in vitro ageing does not fully replicate the biological complexity of the oral cavity. Therefore, these periods represent analytical groupings reflecting material durability in in vitro settings, rather than direct predictors of clinical longevity in complex oral conditions.^27^

Data from the nine included papers revealed 20 biomodifiers, categorized into three main groups, and 51 experimental setups (Figure 4). In these setups, μTBS was the most frequently reported bond test, in addition to being the most adopted testing method in the included reviews. This can easily be explained by being an ideal test for assessing the durability of composite-resin restorations and being widely regarded as the best surrogate measure of dentin bond retention.^28^ Additionally, the most commonly reported time points for bond strength testing were immediately after application and at 12 months (Figure 3). This can easily be explained, as 12 months of aging provides an optimal timeframe for assessing resin-dentin bond durability, simulating long-term degradation mechanisms observed in clinical settings.^29^ Also, thermocycling was the most frequently reported aging method after water storage, as it has proven to cause significant degradation of the adhesive interface and reduce bond strength compared to other aging methods.^30^ This makes it a valuable method for evaluating the long-term durability of adhesive systems.

Furthermore, the long-term durability and success of resin–dentin bonds strongly rely on optimal conditions, which are often difficult to achieve, on both sides of the hybrid layer. To address this, various strategies including natural, chemical, and physical approaches target complementary aspects of the resin-dentin interface. Concerning natural and chemical agents, they act mainly at the molecular level and aim to reinforce the collagen network and reduce enzymatic degradation primarily mediated by MMP-2 and MMP-9, thereby addressing one of the fundamental biological pathways of bond failure.^31^ In contrast, physical agents, act on dentinal surface properties and aim to achieve durable bonds through effective infiltration of adhesives into the dentin. For this, the wettability of the dentin surface is also a critical factor, as it directly influences the ability of adhesive monomers to spread and penetrate into the collagen network.

### Natural agents

It was observed that all natural approaches exhibited either a beneficial effect or no effect on the resin-dentin bond. Grape seed extract demonstrated superior results among polyphenol-rich extracts when used as a pretreatment. This is attributed to its high concentration of proanthocyanidins (PACs), which have been shown to inhibit MMP-2, enhance viscoelastic properties, and improve the surface characteristics of dentin.^32^ However, another highly PAC-rich agent, cocoa seed extract, did not improve bond strength in this review. This can be explained by the fact that CSE contains relatively low content in highly condensed PACs in comparison to the GSE.^11^ Although, recent findings have shown that CSE holds significant potential in reducing enzymatic activity and sealing the resin-dentin interface when used as B-type dimers and trimers under optimized extraction conditions.^33^ Recent studies have shown that other plant extracts used as root canal irrigants with enhanced antibiofilm activity, such as psidium cattleianum, have proved to be highly concentrated in PACs.^34^^,^^35^ Further investigation into their dentin biomodification properties could be beneficial, as it may broaden the scope of their applications. Nevertheless, despite their availability and biocompatibility, the main challenge of PACs lies in their poor stability, which hampers standardization, limits reproducibility and restricts their clinical use.^36^ Alternatively, riboflavin (vitamin B2) is widely recognized for its cross-linking capabilities. Although its dental application remains investigational, riboflavin shows promise for future formulations, supported by its established FDA approval for ophthalmic use in corneal cross-linking.^37^ It also demonstrates dual efficacy when induced by UVA light, inactivating MMP-9 and enhancing the durability of the hybrid layer.^38^ Another natural marine-derived extract, chitosan, has also shown promise as a biomodification solution, effectively preserving bond durability when applied in the etch-and-rinse technique. Although primarily recognized for its chelating properties, chitosan’s functionality has recently been expanded by incorporating mineralization-promoting and anti-inflammatory effects, which together modulate dentin and protect inflamed dentin-pulp complex. Emerging trends include innovative formulations that offer both regenerative and antibacterial effects, such as peptide microspheres with carboxymethyl chitosan or chitosan loaded with calcium phosphate nanoclusters.^39^^,^^40^

### Physical agents

Physical biomodification approaches have been extensively studied in this overview. Non-thermal atmospheric plasma, a partially ionized gas generated at atmospheric pressure, produces reactive species that can enhance dentin wettability, allowing for better adhesive infiltration.^41^ While all reviews agree on the positive effects of NTAP on dentin-resin durability, one study highlights that helium-based NTAP outperforms argon-based NTAP in long-term outcomes.^18^ This superiority may be attributed to helium’s higher ionization energy, which generates a greater concentration of reactive species, leading to more effective oxidation of the dentin surface and consequently increasing wettability.^41^ Despite its promising effects, the clinical translation of NTAP is limited by costly equipment, workflow and chairside integration challenges, and potential tissue damage, all of which hinder its routine use in restorative practice.^42^ Other physical pretreatments that exhibited significant improvement on bond strength are scrubbing and air abrasion. Findings from a recent meta-analysis endorsed the active application of adhesives on dentin in both etch-and-rinse and self-etch modes, as it improves solvent evaporation and resin infiltration.^43^ Also, roughening the dentin surface through air abrasion improves micromechanical interlocking, enhances wettability, and removes residues, all of which strengthen bonding. However, the latter is influenced by particle size and air stream pressure, emphasizing the need for precautions during use.^44^^,^^45^ Conversely, the pooled results for LASER yielded a contrasting outcome. While one study^25^ concluded a decrease in bond strength, the other^19^ observed an increase in shear bond strength, both following LASER pretreatment. Three possible explanations may account for these differences. First, the variation could stem from the fact that it was tested across a variety of experimental designs as the first review included multiple bond strength tests, while the second focused solely on SBS (Figure 4). Second, the type of erbium LASER used might also be a factor, as the second review separated the subgroups based on different LASER types. Lastly, the adhesive type may play a role; 3M ESPE adhesives were found to increase SBS in the Er:YAG group, while Ivoclar Vivadent adhesives enhanced SBS in the Er,Cr:YSGG group. Combined or separate, these factors suggest that further research is needed to fully understand their impact. Beyond these reported agents, high-intensity focused ultrasound (HIFU) represents a novel, energy-based physical strategy worthy of exploration. Although its dental applications remain experimental, HIFU has demonstrated promising potential to enhance dentin adhesion through smear layer removal and dentin remineralization.^46^

### Chemical agents

Unlike the previously discussed categories, chemical agents have predominantly led to negative outcomes, with only few agents with positive effect on bond strength. One of these exceptions is the ethanol-wet technique, which reduces water sorption, inhibits protease activity by inactivating MMPs, and increases interfibrillar spaces, thereby enhancing adhesive impregnation.^47^ A recent study explored a potentially synergistic effect of ethanol wet-bonding and EGCG offering a promising strategy to enhance dentin bonding durability. Although tested on primary teeth, further research on permanent teeth may expand these benefits.^48^ Another notable synergistic combination that was presented as a result in this overview is the use of DMSO as a solvent to effectively incorporate natural plant extracts. DMSO containing pretreatments showcase a unique ability to simultaneously modify both biological and resin components. This blend enhances both dentin wettability and monomer conversion, especially in the deeper hybrid layer, improving infiltration and resulting in stronger resin-dentin bonding.^49^ Moreover, investigating the effect of DMSO combined with other natural agents on bonding to sound dentin may be worth exploring, as a recent study found that eroded dentin pretreated with DMSO and riboflavin achieved bond strength, nanoleakage, and crosslinking rates similar to sound dentin, outperforming untreated eroded dentin.^50^ On the other hand, both NaOCl and HOCl were associated with negative effects, with NaOCl resulting in lower bonding strength. Therefore, when necessary, HOCl could still be recommended as a deproteinizing agent before using self-etch adhesives, with two-step self-etch adhesives offering more reliable bonding. Although the effect may not be significant, some dentin desensitizers have been found to negatively impact adhesion. This might be due to differences in mechanisms of action as well as the wide variety of desensitizers available on the market.^25^^,^^51^ It is safe to concur with Li et al^51^ that the choice of desensitizer should prioritize both bond strength and its effectiveness in relieving dentinal sensitivity after preparation.

### Strengths and limitations

This umbrella review critically evaluated the limitations of dentin biomodification agents through a comparative perspective. To the best of our knowledge, this is the first time the influence of the various existing biomodifiers on the resin-dentin bond durability was systematically analysed and synthesized, marking a significant step forward in the field of restorative dentistry and contributing to Sustainable Development Goal 3 by promoting health. However, it’s important to acknowledge that this work has some limitations that should be considered. In the interest of transparency, this study overviewed all biomodification agents explored by the included SRs, including the adhesive strategies and aging durations employed, offering insight on current research practices. However, outcome data on bond strength were unavailable for some agents, highlighting where further investigation is needed. Also, the considerable heterogeneity in testing methods as presented in Figure 4, outcome metrics (MD, SMD), statistical modelling and divergent effect directions across the included meta-analyses prevented a pooled quantitative analysis of data on bond strength. Moreover, the reviews assessed a wide range of biomodifiers with distinct mechanisms of action, further complicating comparability. For these reasons, a structured qualitative synthesis was undertaken instead, allowing for a comprehensive overview of current evidence. However, future work incorporating in-vivo and clinical studies are needed to complement these findings and to support more directly applicable clinical recommendations.

## Conclusions

Based on the retrieved data for this umbrella review, natural agents top the three categories of biomodifiers. While not yet approved for clinical dental use, they are all considered without harm to adhesion with some being considerably beneficial over time, mainly polyphenol-rich extracts, chitosan and induced riboflavin. Among physical techniques, NTAP, scrubbing, and air abrasion effectively enhance the resin–dentin bond; however, only scrubbing and air abrasion are both affordable and readily applicable in daily practice. In contrast, chemical approaches, particularly NaOCl and HOCl, are not recommended as they compromise bond durability. Future strategies combining natural and physical agents may offer more durable and clinically reliable resin-dentin bond through synergistic effects.

## Authors contributions

*El Alaoui Nihal:* Conceptualization; Methodology; Formal analysis; Investigation; Data Curation; Visualization; Writing – original draft; Writing – review & editing. *Chala Sanaa:* Conceptualization; Methodology; Validation; Writing – review & editing; Supervision. *Ghoul Sonia:* Conceptualization; Methodology; Investigation; Validation; Resources; Writing – review & editing; Supervision; Project Administration.

## Conflict of interest

None disclosed.

## Appendix Supplementary materials

Image, application 1

Image, application 2

## Footnotes

Supplementary material associated with this article can be found in the online version at doi:10.1016/j.identj.2026.109446.

## References

[1] Pfeifer C.S., Lucena F.S., Logan M.G., Nair D., Lewis S.H. (2024). Current approaches to produce durable biomaterials: trends in polymeric materials for restorative dentistry applications. Dent Mater Off Publ Acad Dent Mater.

[2] Zhang Z., Beitzel D., Mutluay M., Tay F.R., Pashley D.H., Arola D. (2015). On the durability of resin-dentin bonds: identifying the weakest links. Dent Mater Off Publ Acad Dent Mater.

[3] AL-Ibrahim I., Shono N., Al-Saud L., Al-Nahedh H. (2025). Five years of restorative resin-based composite advancements: a narrative review. BMC Oral Health.

[4] Schmalz G., Schwendicke F., Hickel R., Platt J.A. (2024). Alternative direct restorative materials for dental amalgam: a concise review based on an FDI policy statement. Int Dent J.

[5] Ferracane J.L. (2024). A historical perspective on dental composite restorative materials. J Funct Biomater.

[6] Pashley D.H., Pashley E.L., Carvalho R.M., Tay F.R. (2002). The effects of dentin permeability on restorative dentistry. Dent Clin North Am.

[7] Melo M.A.S., Garcia I.M., Mokeem L. (2023). Developing bioactive dental resins for restorative dentistry. J Dent Res.

[8] Grawish M.E., Grawish L.M., Grawish H.M. (2022). Demineralized dentin matrix for dental and alveolar bone tissues regeneration: an innovative scope review. Tissue Eng Regen Med.

[9] Betancourt D.E., Baldion P.A., Castellanos J.E. (2019). Resin-dentin bonding interface: mechanisms of degradation and strategies for stabilization of the hybrid layer. Int J Biomater.

[10] Mokeem L.S., Garcia I.M., Melo M.A. (2023). Degradation and failure phenomena at the dentin bonding interface. Biomedicines.

[11] Aguiar T.R., Vidal C.M.P., Phansalkar R.S. (2014). Dentin biomodification potential depends on polyphenol source. J Dent Res.

[12] da Silva I.S.P., Bordini E.A.F., Bronze-Uhle E.S. (2024). Photo-crosslinkable hydrogel incorporated with bone matrix particles for advancements in dentin tissue engineering. J Biomed Mater Res A.

[13] Bedran-Russo A.K., Pauli G.F., Chen S.N. (2014). Dentin biomodification: strategies, renewable resources and clinical applications. Dent Mater Off Publ Acad Dent Mater.

[14] Van Meerbeek B., Yoshihara K., Van Landuyt K., Yoshida Y., Peumans M. (2020). From Buonocore’s pioneering acid-etch technique to self-adhering restoratives: a status perspective of rapidly advancing dental adhesive technology. J Adhes Dent.

[15] Pérez-Bracchiglione J., Meza N., Bangdiwala S.I. (2022). Graphical representation of overlap for OVErviews: GROOVE tool. Res Synth Methods.

[16] Pieper D., Antoine S.L., Mathes T., Neugebauer E.A.M., Eikermann M. (2014). Systematic review finds overlapping reviews were not mentioned in every other overview. J Clin Epidemiol.

[17] Whiting P., Savović J., Higgins J.P.T. (2016). ROBIS: a new tool to assess risk of bias in systematic reviews was developed. J Clin Epidemiol.

[18] Stasic J.N., Pficer J.K., Milicic B., Puač N., Miletic V. (2021). Effects of non-thermal atmospheric plasma on dentin wetting and adhesive bonding efficiency: systematic review and meta-analysis. J Dent.

[19] Wang J., Chen S., Wu Y. (2024). The influence of erbium laser pretreatment on dentin shear bond strength and bond failure modes: a systematic review and network meta-analysis. J Adhes Dent.

[20] Zhang Z., Li K., Yang H., Yu J., Huang C. (2022). The influence of dimethyl sulfoxide on resin-dentin bonding: a systematic review. Int J Adhes Adhes.

[21] Awad M.M., Alhalabi F., Alshehri A. (2021). Effect of non-thermal atmospheric plasma on micro-tensile bond strength at adhesive/dentin interface: a systematic review. Materials.

[22] Eusufzai S.Z., Barman A., Jamayet N.B. (2023). Effects of riboflavin collagen crosslinker on dentin adhesive bonding efficiency: a systematic review and meta-analysis. Mater Basel Switz.

[23] Alves LVGL, Cerqueira N.M., Candemil A.P., Faria-e-Silva A.L., Sousa-Neto M.D., Souza-Gabriel A.E. (2024). Does dentin pretreatment with chitosan improve the bond strength of restorative material? A systematic review and meta-analysis of in vitro studies. Int J Adhes Adhes.

[24] Anumula L., Ramesh S., Kolaparthi V.S.K. (2022). Role of natural cross linkers in resin-dentin bond durability: a systematic review and meta-analysis. Mater Basel Switz.

[25] Hardan L., Bourgi R., Kharouf N. (2021). Bond strength of universal adhesives to dentin: a systematic review and meta-analysis. Polymers.

[26] Alshaikh K.H., Hamama H.H.H., Mahmoud S.H. (2018). Effect of smear layer deproteinization on bonding of self-etch adhesives to dentin: a systematic review and meta-analysis. Restor Dent Endod.

[27] Rêgo H.M., Alves T.S., Bresciani E., Niu L.N., Tay F.R., Pucci C.R. (2016). Can long-term dentine bonding created in real life be forecasted by parameters established in the laboratory?. Sci Rep.

[28] Van Meerbeek B., Peumans M., Poitevin A. (2010). Relationship between bond-strength tests and clinical outcomes. Dent Mater.

[29] Hosaka K., Nishitani Y., Tagami J. (2009). Durability of resin-dentin bonds to water- vs. ethanol-saturated dentin. J Dent Res.

[30] Teixeira G.S., Pereira G.K.R., Susin A.H. (2021). Aging methods—an evaluation of their influence on bond strength. Eur J Dent.

[31] Barbosa C de B., Monici Silva I., de Cena J.A., Stefani C.M., Dame-Teixeira N. (2023). Presence of host and bacterial-derived collagenolytic proteases in carious dentin: a systematic review of ex vivo studies. Front Cell Infect Microbiol.

[32] Mendes T., Pascoal S., Estellita M., Lemos M., Silva P., Mendonça J. (2022). Effect of grape seed extract on stability of restorations with composites resin: a systematic review and meta-analysis. Int J Adhes Adhes.

[33] Sayed M., Jing S., Chen S.N., Pauli G.F., Bedran-Russo A.K. (2025). A dual action approach of B-type proanthocyanidins for silencing enzymatic activity and sealing natural caries-affected dentin-resin interfaces. J Dent.

[34] Diouchi J., Touré B., Ghoul S. (2024). Antibiofilm efficacy of plant extracts as root canal irrigants in endodontics: a systematic literature review. Front Dent Med.

[35] González-Silva N., Nolasco-González Y., Aguilar-Hernández G. (2022). Ultrasound-assisted extraction of phenolic compounds from psidium cattleianum leaves: optimization using the response surface methodology. Molecules.

[36] Alania Y., Reis-Havlat M., Jing S.X. (2025). Structure-activity relationships of A-and B-type proanthocyanidins in long-term dentin biomodification and biocompatibility. J Dent.

[37] Belin M.W., Lim L., Rajpal R.K., Hafezi F., Gomes J.A.P., Cochener B. (2018). Corneal cross-linking: current USA status: report from the cornea society. Cornea.

[38] Cova A., Breschi L., Nato F. (2011). Effect of UVA-activated riboflavin on dentin bonding. J Dent Res.

[39] Wang L., Ou Y., Wang J., Ding L., Han S., Zhang L. (2025). Two-stepped pH-responsive peptide microsphere/carboxymethyl chitosan complex: enhanced protection of an inflamed dentin-pulp complex. J Mater Chem B.

[40] Liu X., Zhang S., Zheng H. (2025). Quaternary ammonium chitosan film loading ultrasmall calcium phosphate nanoclusters for biomimetic mineralization with antibacterial ability. Int J Biol Macromol.

[41] Seo Y., Mohamed A.A., Woo K., Lee H., Koo J., Kim K. (2010). Comparative studies of atmospheric pressure plasma characteristics between He and Ar working gases for sterilization. IEEE Trans Plasma Sci - IEEE TRANS PLASMA SCI.

[42] Green M., Linkous C., Strat N., Valdebran M. (2023). Potential uses of nonthermal atmospheric pressure technology for dermatologic conditions in children. Cutis.

[43] Carvalho G.L.M., Moreira P.M., Carneiro B.T. (2025). Impact of active vs. passive application of dental adhesives on bond strength to dentin and enamel: a systematic review and meta-analysis of in vitro studies. Jpn Dent Sci Rev.

[44] Reis A., Feitosa V.P., Chibinski A.C., Favoreto M.W., Gutierrez M.F., Loguercio A.D. (2024). Biomimetic restorative dentistry: an evidence-based discussion of common myths. J Appl Oral Sci.

[45] Lima V., Soares K., Caldeira V., Faria-e-Silva A., Loomans B., Moraes R. (2020). Airborne-particle abrasion and dentin bonding: systematic review and meta-analysis. Oper Dent.

[46] Tran M.D., Ngo H., Fawzy A. (2024). High-intensity focused ultrasound in dentistry: a literature review. Int Dent J.

[47] Telles C.C., da C., Basting R.T., Bridi E.C., França F.M.G., do Amaral F.L.B., Basting R.T. (2023). Wet-bonding technique with ethanol may reduce protease activity in dentin-resin interface following application of universal adhesive system. J Clin Exp Dent.

[48] Yu J., Zhao Y., Shen Y. (2022). Enhancing adhesive-dentin interface stability of primary teeth: from ethanol wet-bonding to plant-derived polyphenol application. J Dent.

[49] Stape T.H.S., Mutluay M.M., Tjäderhane L., Uurasjärvi E., Koistinen A., Tezvergil-Mutluay A. (2021). The pursuit of resin-dentin bond durability: simultaneous enhancement of collagen structure and polymer network formation in hybrid layers. Dent Mater Off Publ Acad Dent Mater.

[50] Wendlinger M., Cochinski G.D., Aguiar Moreira P.H., Cardenas A.F.M., de Siqueira F.S.F., Stape T.H.S., Tezvergil-Mutluay A., Loguercio A.D. (2025). Effect of proanthocyanidin and riboflavin associated with dimethyl sulfoxide in eroded dentine: six-year in vitro evaluation. Dent Mater.

[51] Li J., Hua F., Xu P., Huang C., Yang H. (2021). Effects of desensitizers on adhesive-dentin bond strength: a systematic review and meta-analysis. J Adhes Dent.

