# Supplementary material for: Effect of Dentin Biomodification on the Survival of Resin Composite Restorations: An Umbrella Review
Source: Int Dent J. 2026 Feb 18;76(2):109446. doi: 10.1016/j.identj.2026.109446 (PMC12933814; doi:10.1016/j.identj.2026.109446)
Supplement: Supplementary file 1 [file mmc1.docx]

**Table A.1 Search string for PubMed database search**

Search performed on October 6^th^, 2024

| Search string | | Filters | Number of articles |
| --- | --- | --- | --- |
| #1 | (“dentin” OR “dentine”) | By article type: systematic reviews | 39 |
| #2 | ("biomodif*" OR "bio-modif*" OR "pretreat*" OR "pre-treat*" OR "Matrix Metalloproteinases"[Mesh] OR "Matrix Metalloproteinase Inhibitors"[Mesh] OR "matrix metalloproteinases inhibitors" OR "matrix metalloproteinases" OR "cross link*" OR "cross-link*" OR "crosslink*" OR "UVA" OR "riboflavin" OR "flavonoid" OR "polyphenols" OR "proanthocyanidin" OR "genipin" OR "chitosan" OR "citric acid" OR "chlorhexidine" OR "CHX" OR "glutaraldehyde" OR "cpp-acp" OR "carbodiimide" OR "EDC" OR "EGCG" OR "quaternary ammonium" OR "edta") |  |  |
| #3 | ("bond strength" OR “survival” OR "bond durability" OR "tensile strength" OR "shear strength" OR "microtensile strength" OR "microshear strength" OR “micro-tensile strength” OR “micro-shear strength”) |  |  |
| #1 AND #2 AND #3 | |  |  |

**Table A.2 Search string for Scopus database search**

Search performed on October 6^th^, 2024

| Search string | | Filters | Number of articles |
| --- | --- | --- | --- |
| #1 | ("dentin" OR "dentine") | By article subject:   - Dentistry - Material science - Medicine   By type: review | 395 |
| #2 | ("biomodif*" OR "bio modif" OR "pretreat*" OR "pre-treat*" OR "matrix metalloproteinases inhibitors" OR "matrix metalloproteinases" OR "MMP*" OR "cross link*" OR "crosslink*" OR "UVA" OR "riboflavin" OR "flavonoid" OR "polyphenols" OR "proanthocyanidin" OR "genipin" OR "chitosan" OR "citric acid" OR "chlorhexidine" OR "CHX" OR "glutaraldehyde" OR "cpp-acp" OR "carbodiimide" OR "EDC" OR "EGCG" OR "quaternary ammonium" OR "edta") |  |  |
| #3 | ("bond strength" OR "bond durability" OR “survival” OR "tensile strength" OR "shear strength" OR "microtensile strength" OR "microshear strength" OR "micro-tensile strength" OR "micro-shear strength") |  |  |
| #4 | (“systematic”) |  |  |
| #1 AND #2 AND #3 AND #4 | |  |  |

**Table A.3 Search string for Web of Science Core Collection** **database search**

Search performed on October 20^th^, 2024

| Search string | | Filters | Number of articles |
| --- | --- | --- | --- |
| #1 | (“dentin” OR “dentine”) | By type: review article | 52 |
| #2 | ("biomodif*" OR "bio-modif*" OR "pretreat*" OR "pre-treat*" OR "Matrix Metalloproteinases"[Mesh] OR "Matrix Metalloproteinase Inhibitors"[Mesh] OR "matrix metalloproteinases inhibitors" OR "matrix metalloproteinases" OR "cross link*" OR "cross-link*" OR "crosslink*" OR "UVA" OR "riboflavin" OR "flavonoid" OR "polyphenols" OR "proanthocyanidin" OR "genipin" OR "chitosan" OR "citric acid" OR "chlorhexidine" OR "CHX" OR "glutaraldehyde" OR "cpp-acp" OR "carbodiimide" OR "EDC" OR "EGCG" OR "quaternary ammonium" OR "edta") |  |  |
| #3 | ("bond strength" OR “survival” OR "bond durability" OR "tensile strength" OR "shear strength" OR "microtensile strength" OR "microshear strength" OR “micro-tensile strength” OR “micro-shear strength”) |  |  |
| #4 | (“systematic”) |  |  |
| #1 AND #2 AND #3 AND #4 | |  |  |

**Table A.4 Eligibility criteria based on PICOS elements**

| PICOS elements | Include | Exclude |
| --- | --- | --- |
| Population | Resin-composites restorations of dentin from adult human teeth | - Materials other than composite-resins e.g. Sealants, luting cements, fiber posts etc. - Non-human teeth - Deciduous teeth - Teeth with structural anomalies |
| Intervention | Application of biomodifiers on dentin surface prior to restoration | Biomodification agents incorporated in adhesives or composite-resins |
| Comparators | Resin-composite restorations with no prior dentin pretreatment | NA |
| Outcome | Bond durability of resin-composites | NA |
| Study design | Systematic reviews of *in vitro* studies, with or without meta-analysis | Narrative reviews, Scoping reviews, Bibliometric analysis, Systematic review of clinical studies, Systematic review of methods studies, Systematic reviews of animal studies |

**NA: Not Applicable.**
