# Supplementary material for: Effect of Dentin Biomodification on the Survival of Resin Composite Restorations: An Umbrella Review
Source: Int Dent J. 2026 Feb 18;76(2):109446. doi: 10.1016/j.identj.2026.109446 (PMC12933814; doi:10.1016/j.identj.2026.109446)

**Table B.1 CCA matrix**

| **MATRIX OF EVIDENCE** | |  |  |  |  |  |  |  |  |
| --- | --- | --- | --- | --- | --- | --- | --- | --- | --- |
| **Primary Studies** |  |  |  | **Systematic Reviews** | | |  |  |  |
| **Study ID** | **Alshaikh et al. (2018)** | **Alves and al. (2024)** | **Anumula et al. (2022)** | **Awad et al. (2021)** | **Eusufzai et al. (2023)** | **Hardan et al. (2021)** | **Stasic and al. (2021)** | **Wang and al. (2024)** | **Zhang et al. (2022)** |
| Taniguchi et al. 2009 | 1 |  |  |  |  |  |  |  |  |
| Kunawarote et al. 2010 | 1 |  |  |  |  |  |  |  |  |
| Cecchin et al. 2010 | 1 |  |  |  |  |  |  |  |  |
| Farina et al. 2011 | 1 |  |  |  |  |  |  |  |  |
| Ozturk et al. 2004 | 1 |  |  |  |  |  |  |  |  |
| Prasansuttiporn et al. 2011 | 1 |  |  |  |  |  |  |  |  |
| Kunawarote et al. 2011 | 1 |  |  |  |  |  |  |  |  |
| Prasansuttiporn et al. 2012 | 1 |  |  |  |  |  |  |  |  |
| Sacramento et al. 2011 | 1 |  |  |  |  |  |  |  |  |
| Gu et al. 2019 |  | 1 |  |  |  |  |  |  |  |
| Nivedita et al. 2019 |  | 1 |  |  |  |  |  |  |  |
| Vasei et al. 2021 |  | 1 |  |  |  |  |  |  |  |
| Shan et al. 2022 |  | 1 |  |  |  |  |  |  |  |
| Yu et al. 2021 |  | 1 |  |  |  |  |  |  |  |
| Baena et al.2020 |  | 1 |  |  |  | 1 |  |  |  |
| Ziotti et al. 2022 |  | 1 |  |  |  |  |  |  |  |
| Zhou et al. 2022 |  | 1 |  |  |  |  |  |  |  |
| Paschoini et al. 2021 |  | 1 |  |  |  |  |  |  |  |
| Mohamed et al. 2020 |  | 1 |  |  |  | 1 |  |  |  |
| Zidan et al. 2019 |  | 1 |  |  |  |  |  |  |  |
| Castellan et al. 2013 |  |  | 1 |  |  |  |  |  |  |
| Al-Ammar et al. 2009 |  |  | 1 |  |  |  |  |  |  |
| Castellan et al. 2010 |  |  | 1 |  |  |  |  |  |  |
| Epasinghe et al. 2012 |  |  | 1 |  |  |  |  |  |  |
| Monteiro et al. 2013 |  |  | 1 |  |  |  |  |  |  |
| Zheng et al. 2015 |  |  | 1 |  |  |  |  |  |  |
| Neri et al. 2016 |  |  | 1 |  |  |  |  |  |  |
| Gerhardt et al. 2016 |  |  | 1 |  |  |  |  |  |  |
| Zheng et al. 2017 |  |  | 1 |  |  |  |  |  |  |
| Chu et al. 2019 |  |  | 1 |  |  |  |  |  |  |
| Gajjela et al. 2017 |  |  | 1 |  | 1 |  |  |  |  |
| Hass et al. 2016 |  |  | 1 |  | 1 |  |  |  |  |
| Venigella et al. 2016 |  |  | 1 |  |  |  |  |  |  |
| Li et al. 2018 |  |  | 1 |  |  |  |  |  | 1 |
| Hirata et al.2016 |  |  |  | 1 |  |  | 1 |  |  |
| Abreu et al. 2016 |  |  |  | 1 |  |  | 1 |  |  |
| Hirata et al.2015 |  |  |  | 1 |  |  |  |  |  |
| Han et al. 2014 |  |  |  | 1 |  |  | 1 |  |  |
| Han et al.2019 |  |  |  | 1 |  |  | 1 |  |  |
| Ayres et al. 2018 |  |  |  | 1 |  | 1 | 1 |  |  |
| Ayres et al. 2018 (b) |  |  |  | 1 |  | 1 | 1 |  |  |
| Zhu et al. 2018 |  |  |  | 1 |  |  | 1 |  |  |
| Zhu et al. 2018 (b) |  |  |  | 1 |  |  | 1 |  |  |
| Kim et al. 2016 |  |  |  | 1 |  |  | 1 |  |  |
| Dong et al. 2015 |  |  |  | 1 |  |  | 1 |  |  |
| Dong et al. 2013 |  |  |  | 1 |  |  | 1 |  |  |
| Kitts et al. 2010 |  |  |  | 1 |  |  |  |  |  |
| Hashem et al. 2021 |  |  |  |  | 1 |  |  |  |  |
| Daood et al. 2014 |  |  |  |  | 1 |  |  |  |  |
| Abunawareg et al. 2017 |  |  |  |  | 1 |  |  |  |  |
| Daood et al. 2018 |  |  |  |  | 1 |  |  |  |  |
| Daood et al. 2020 |  |  |  |  | 1 |  |  |  |  |
| Chiang et al. 2013 |  |  |  |  | 1 |  |  |  |  |
| Hass et al. 2016 (b) |  |  |  |  | 1 |  |  |  |  |
| Cova et al. 2011 |  |  |  |  | 1 |  |  |  |  |
| Fawzy et al. 2013 |  |  |  |  | 1 |  |  |  |  |
| Fawzy et al. 2012 |  |  |  |  | 1 |  |  |  |  |
| Fu et al. 2020 |  |  |  |  | 1 |  |  |  |  |
| Venigalla et al. 2016 |  |  |  |  | 1 |  |  |  |  |
| Daood et al. 2020 (b) |  |  |  |  | 1 |  |  |  |  |
| Abuelenain et al. 2018 |  |  |  |  | 1 |  |  |  |  |
| Daood et al. 2021 |  |  |  |  | 1 |  |  |  |  |
| Flurry et al. 2017 |  |  |  |  |  | 1 |  |  |  |
| Sutil et al. 2017 |  |  |  |  |  | 1 |  |  |  |
| Bacelar-Sá et al. 2017 |  |  |  |  |  | 1 |  |  |  |
| Lima et al. 2018 |  |  |  |  |  | 1 |  |  |  |
| Kaynar et al. 2020 |  |  |  |  |  | 1 |  |  |  |
| Kusdemir et al. 2015 |  |  |  |  |  | 1 |  |  |  |
| Paulose et al. 2017 |  |  |  |  |  | 1 |  |  |  |
| Luong et al. 2020 |  |  |  |  |  | 1 |  |  |  |
| Siso et al. 2016 |  |  |  |  |  | 1 |  |  |  |
| Bravo et al. 2017 |  |  |  |  |  | 1 |  |  |  |
| Chaharom et al. 2019 |  |  |  |  |  | 1 |  |  | 1 |
| Giocomini et al. 2020 |  |  |  |  |  | 1 |  |  |  |
| Peng et al. 2020 |  |  |  |  |  | 1 |  |  |  |
| Shadman et al. 2018 |  |  |  |  |  | 1 |  |  |  |
| Tekçe et al. 2016 |  |  |  |  |  | 1 |  |  |  |
| Vivanco et al. 2020 |  |  |  |  |  | 1 |  |  |  |
| Zenobi et al. 2017 |  |  |  |  |  | 1 |  |  |  |
| Ahn et al. 2014 |  |  |  |  |  | 1 |  |  |  |
| Comba et al. 2019 |  |  |  |  |  | 1 |  |  |  |
| Sellan et al. 2020 |  |  |  |  |  | 1 |  |  |  |
| Shadman et al. 2019 |  |  |  |  |  | 1 |  |  |  |
| Silva et al. 2019 |  |  |  |  |  | 1 |  |  |  |
| Silva et al. 2016 |  |  |  |  |  | 1 |  |  |  |
| Rechmann et al. 2017 |  |  |  |  |  | 1 |  |  |  |
| Trevelin et al. 2019 |  |  |  |  |  | 1 |  |  |  |
| Yazici et al. 2016 |  |  |  |  |  | 1 |  |  |  |
| Irmak et al. 2018 |  |  |  |  |  | 1 |  |  |  |
| Cecchin et al. 2018 |  |  |  |  |  | 1 |  |  |  |
| Cangul et al. 2020 |  |  |  |  |  | 1 |  |  |  |
| Chen et al. 2020 |  |  |  |  |  | 1 |  |  |  |
| Paulose et al. 2018 |  |  |  |  |  | 1 |  |  |  |
| Bravo et al. 2017 (b) |  |  |  |  |  | 1 |  |  |  |
| Jang et al. 2018 |  |  |  |  |  | 1 |  |  |  |
| Guarda et al. 2020 |  |  |  |  |  | 1 |  |  |  |
| Thanatvarakorn et al. 2016 |  |  |  |  |  | 1 |  |  |  |
| Thanatvarakorn et al. 2018 |  |  |  |  |  | 1 |  |  |  |
| Cha et al. 2016 |  |  |  |  |  | 1 |  |  |  |
| Zhang et al. 2020 |  |  |  |  |  | 1 |  |  | 1 |
| Stasic et al. 2019 |  |  |  |  |  |  | 1 |  |  |
| Chen et al. 2013 |  |  |  |  |  |  | 1 |  |  |
| Dong et al. 2014 |  |  |  |  |  |  | 1 |  |  |
| Ritts et al. 2010 |  |  |  |  |  |  | 1 |  |  |
| Imiolczyk et al. 2018 |  |  |  |  |  |  | 1 |  |  |
| Ayres et al.2017 |  |  |  |  |  |  | 1 |  |  |
| Al Habdan et al. 2021 |  |  |  |  |  |  |  | 1 |  |
| Al-Jeaidi et al. 2020 |  |  |  |  |  |  |  | 1 |  |
| Alkhudhairy et al. 2020 |  |  |  |  |  |  |  | 1 |  |
| Alkhudhairy et al. 2020 (b) |  |  |  |  |  |  |  | 1 |  |
| Al-Khureif et al. 2020 |  |  |  |  |  |  |  | 1 |  |
| Almutairi et al. 2021 |  |  |  |  |  |  |  | 1 |  |
| Altunsoy et al. 2014 |  |  |  |  |  |  |  | 1 |  |
| Bahrololoomi et al. 2017 |  |  |  |  |  |  |  | 1 |  |
| Beer et al. 2011 |  |  |  |  |  |  |  | 1 |  |
| Bertrand et al. 2006 |  |  |  |  |  |  |  | 1 |  |
| Brulat et al. 2008 |  |  |  |  |  |  |  | 1 |  |
| Capa et al. 2010 |  |  |  |  |  |  |  | 1 |  |
| Ceballo et al. 2002 |  |  |  |  |  |  |  | 1 |  |
| Chemaly et al. 2022 |  |  |  |  |  |  |  | 1 |  |
| Chou et al. 2009 |  |  |  |  |  |  |  | 1 |  |
| Curylofo et al. 2014 |  |  |  |  |  |  |  | 1 |  |
| Cvikl et al. 2011 |  |  |  |  |  |  |  | 1 |  |
| Dilber et al. 2015 |  |  |  |  |  |  |  | 1 |  |
| Dunn et al. 2005 |  |  |  |  |  |  |  | 1 |  |
| Elsahn et al. 2021 |  |  |  |  |  |  |  | 1 |  |
| Garbui et al. 2013 |  |  |  |  |  |  |  | 1 |  |
| Giray et al. 2014 |  |  |  |  |  |  |  | 1 |  |
| Gisler et al. 2012 |  |  |  |  |  |  |  | 1 |  |
| Gurgan et al. 2008 |  |  |  |  |  |  |  | 1 |  |
| Guven et al. 2013 |  |  |  |  |  |  |  | 1 |  |
| Ismatullaev et al. 2020 |  |  |  |  |  |  |  | 1 |  |
| Karadas et al. 2017 |  |  |  |  |  |  |  | 1 |  |
| Jhingan et al. 2015 |  |  |  |  |  |  |  | 1 |  |
| Meriç et al. 2016 |  |  |  |  |  |  |  | 1 |  |
| Nahas et al. 2016 |  |  |  |  |  |  |  | 1 |  |
| Ribeiro et al. 2013 |  |  |  |  |  |  |  | 1 |  |
| Sharafeddin et al. 2022 |  |  |  |  |  |  |  | 1 |  |
| Shirani et al. 2012 |  |  |  |  |  |  |  | 1 |  |
| Shirani et al. 2014 |  |  |  |  |  |  |  | 1 |  |
| Staninec et al. 2006 |  |  |  |  |  |  |  | 1 |  |
| Ustunkol et al. 2015 |  |  |  |  |  |  |  | 1 |  |
| Visuri et al. 1996 |  |  |  |  |  |  |  | 1 |  |
| Vohra et al. 2018 |  |  |  |  |  |  |  | 1 |  |
| Xiong et al. 2022 |  |  |  |  |  |  |  | 1 |  |
| Yazici et al. 2010 |  |  |  |  |  |  |  | 1 |  |
| Tjäderhane et al. 2013 |  |  |  |  |  |  |  |  | 1 |
| Stape et al. 2015 |  |  |  |  |  |  |  |  | 1 |
| Stape et al. 2016 |  |  |  |  |  |  |  |  | 1 |
| Guo et al. 2017 |  |  |  |  |  |  |  |  | 1 |
| Stape et al. 2018 |  |  |  |  |  |  |  |  | 1 |
| Al-Ani et al. 2018 |  |  |  |  |  |  |  |  | 1 |
| Stape et al. 2018 (b) |  |  |  |  |  |  |  |  | 1 |
| Li et al. 2020 |  |  |  |  |  |  |  |  | 1 |
| Saffarpour et al. 2020 |  |  |  |  |  |  |  |  | 1 |

**Figure B.1 Graphical representation of overlap for OVErviews (GROOVE)**


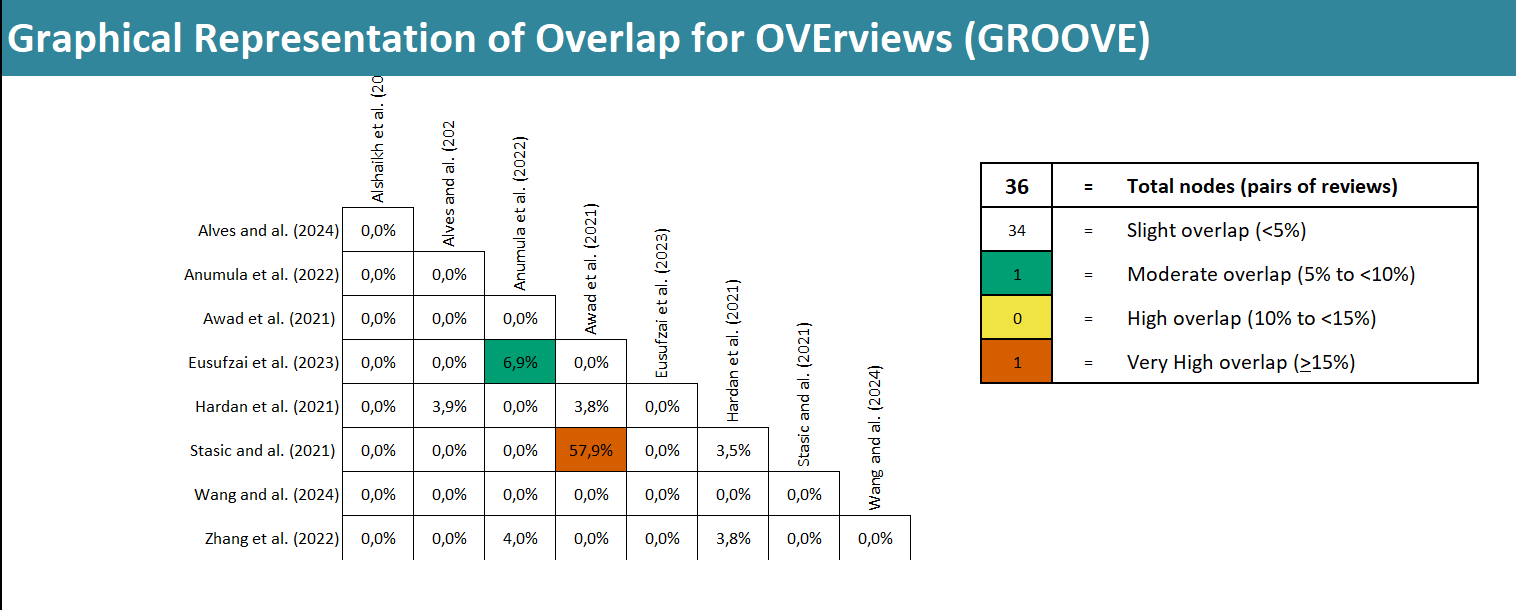

Supplement: Supplementary file 2 [file mmc2.docx]
